# Validation of the condom use self-efficacy scale in Ethiopia

**DOI:** 10.1186/1472-698X-13-22

**Published:** 2013-04-23

**Authors:** Debebe Shaweno, Emebet Tekletsadik

**Affiliations:** 1 Epidemiology and Biostatistics unit, School of Public and Environmental Health, Hawassa University, P.O.Box 1560, Hawassa, Ethiopia; 2 Reproductive Health and Nutrition unit, School of Public and Environmental Health, Hawassa University, P.O.Box 1560, Hawassa, Ethiopia

**Keywords:** Validation, CUSES-E, Hawassa University

## Abstract

**Background:**

The measurement of condom use self-efficacy requires contextually suitable, valid and reliable instruments due to variability of the scale across nations with different cultural and ethnic backgrounds. This study aims to construct a condom use self-efficacy scale suitable to Ethiopia (CUSES-E), based on the original scale developed by Brafford and Beck.

**Methods:**

A cross-sectional study was conducted on a random sample of 492 students at Hawassa University. A self-administered questionnaire containing 28 items from the original scale was used to collect the data. Principal Component Analysis (PCA) with Varimax rotation was used to extract factor structures. Cronbach’s alpha and item-total correlations were used to determine the internal consistency of the scale. The convergent and discriminant validity of the scale was verified using a correlation matrix.

**Results:**

The PCA extracted three factors containing a total of 9-items. The extracted factors were labeled Assertiveness, Fear for partner rejection and Intoxicant Control, with internal consistency coefficients (Cronbach’s alpha) of 0.86, 0.86 and 0.92, respectively. Altogether, the factors explained 77.8% of variance in the items. An evaluation of CUSES-E showed a significantly higher self-efficacy score among students who ever used condoms; P < 0.001. The correlation matrix revealed that all of the convergent correlations were higher than the discriminant ones, providing evidence in support of both types of validity. In the split sample validation, the communalities, factor loadings and factor structure were the same on the analysis on each half and the full data set, suggesting that the new scale is generalizable and replicable.

**Conclusion:**

This study of CUSES using an Ethiopian population found a different dimension to emerge, suggesting that the scale should be validated to local contexts before application. The CUSES-E is valid, reliable and replicable. Therefore, health cadres and researchers in Ethiopia can apply this scale to promote condom utilization to Ethiopian school youths. However, future research to develop a suitable scale (highly valid and reliable) in concordance with the local vernacular using a prior qualitative study is needed.

## Background

Sexual intercourse is the main mode of HIV transmission in Ethiopia, which is mainly driven by young people [1]. Condoms are key components of prevention strategies that individuals can choose to reduce their risks of sexual exposure to HIV [2], however, condom utilization for prevention of HIV transmission requires people to exercise control over their own behavior. Even though individuals acknowledge that safer sex practices reduce risk of HIV infection and posses the required skills, they do not adopt them when they lack a sense of self-efficacy [3].

Self efficacy is a central concept in the application of social cognitive theory to health promotion. According to social cognitive theory, both outcome and efficacy expectations are critical to behavior change [4,5]. Self-efficacy is a belief an individual uses to execute a measure of control over his or her environment [6]. According to self-efficacy theory, people’s beliefs in their personal efficacy can be developed by four main sources of influence [7,8]. The sources are performance accomplishment, vicarious experiences, verbal persuasion and physiological arousal. Performance accomplishment is learning from previous personal experience where one has achieved mastery. Vicarious experience refers to learning by observing the successful social models. Verbal persuasion implies that people who are persuaded verbally that they possess the capabilities to master given activities are likely to mobilize greater effort and sustain it. Finally physiological arousal refers to physical feedback that individuals encounter when attempting a given task [6-8].

The moment occasioned by Bandura’s assertion that self-efficacy could be a useful tool in HIV reduction has facilitated the development of Condom Use Self-Efficacy Scale (CUSES) by Brafford and Beck [8]. The original CUSES consisted of 28 items describing an individual’s feelings of confidence about being able to purchase condoms, put them on and take them off, and negotiate their use with a new sexual partner [9]. Subsequent studies of CUSES showed that individuals with high condom use self-efficacy were more likely to purchase, possess and apply condoms [8-10]. Thus, HIV risk reduction calls for enhancement of interpersonal efficacy rather than simply targeting a specific behavior change.

In Ethiopia, despite the knowledge about its preventive efficacy, utilization among youth is limited even when condoms are readily available [11,12]. Consistent condom use is very low, and is estimated to be around 20% among high school or college students [13]. Lack of access to and low condom use self-efficacy are among the factors believed to be related to inconsistent condom use. In the context of the study area (Hawassa University), lack of access may not be a major problem because the university is located in the metropolitan city where condoms are readily available at kiosks in public areas. However, the problem might be related to fear of acquiring and/or possessing, which involves issues of condom use self-efficacy as noted in a national HIV behavioral surveillance survey in Ethiopia [11].

In order to measure condom use self-efficacy, valid and reliable instruments are necessary. The CUSES developed by Brafford and Beck was as such valid and reliable [14]. With the exception of a single study conducted in Africa (i.e., Ghana), many validation studies of the CUSES have been conducted in populations based in high socioeconomic countries [8,14].

Different validation studies showed the scale to vary markedly among populations with different ethnic and cultural backgrounds [8,10,14]. Bandura puts this heterogeneity of the scale across different socio economic settings as, “There is no all-purpose measure of perceived self-efficacy” [15]. Previous validation studies resulted in different factor solutions with marked difference in the number and types of items loading. For instance, while a study by Barkley and Burns (Florida) resulted in three factor structure leaving 18(64.3%) items unassigned, the study by Asante and Doku (Ghana) resulted in four factor structure leaving 50% of the items unassigned [8,10]. A study by Brien et al left 13 items unassigned [16]. Despite this heterogeneity, some researchers in Africa, including Ethiopia, have utilized non-validated items from the scale in studies investigating self-efficacy as a determinant of condom use [17-19]. The findings of such studies are likely to be flawed, because the cultural suitability of the scale to their context has not been established. While developing a scale with a local vernacular remains the best option to measure self-efficacy adequately, validating and using the existing scale appears to be not only economical but also gives an advantage of comparison with other similar previous studies [10]. This study aims to construct a condom use self-efficacy scale suitable to Ethiopia (CUSES-E), based on the original scale developed by Brafford and Beck.

## Methods

### Condom use self-efficacy scale

This scale was originally developed to assess the confidence of American college students towards condom use. It consisted of 28 items describing an individual’s feelings of confidence about being able to purchase condoms, put them on and take them off, and negotiate their use with a new sexual partner [14]. The later analysis resulted in four subscales: mechanics(items 1,27, 14,22), partner disapproval(items 9,10,16, 17,18), assertive(items 4,5,6) and intoxicants(items 24, 25, 28), and left 13 items unassigned [17]. The subscales had high internal consistencies (Cronbach’s alpha) of 0.78, 0.81, 0.80 and 0.82 respectively [10]. The original scale had adequate reliability (Cronbach’s alpha = 0.91 and a two week test-retest correlation of 0.81) [14].

The items elicit responses using a five-point Likert scale that ranges from ‘strongly disagree’ to ‘strongly agree’. In this study, the responses for each item were scored as 0 = strongly disagree, 1 = disagree, 2 = undecided, 3 = agree and 4 = strongly agree. Before the analysis, 7 negatively worded items were reverse coded. Higher scores indicate greater condom use self-efficacy.

#### Setting and design

A cross-sectional study was conducted in Hawassa University, which is one of the 30 governmental universities in Ethiopia. It is located in southern Ethiopia around 300 kilometers from the capital, Addis Ababa. It enrolls students from all regions of the country and had around 25,000 in-campus students studying in 58 departments at the time of the study, 2012.

The study was conducted among students in a randomly selected 13 sections. A proportional number of participants were randomly selected from each of the selected sections.

#### Sample size

A rule of thumb that the subject-to-item ratio for exploratory factor analysis should be at least 10:1 [20] was considered. Assuming a design effect of 1.6 and a non-response rate of 10%, the calculated sample size was 492.

### Data collection procedure

Class schedules of the randomly selected departments were sought from the respective schools. The course instructors were asked to allow the students to fill out the questionnaire during the first 25 minutes of their lecture time. The selected students were then provided by investigators with an in-class orientation that described the study, how they were selected, and confidentiality issues. Students were also informed that refusal to participate in the study would not have any consequence to the service they get from the university. Students were asked not to include any personal identifiers on the questionnaire. The questionnaires containing all 28 items from the original scale were handed out to the students who agreed to take it. Prior to data collection the protocol was approved by the Institutional Review Board of Hawassa University, College of Medicine and Health Sciences.

### Data analysis

Data were analyzed using SPSS version 20. Levene’s test was used to evaluate equality of variance between groups compared using *t*-test after checking the normality of the distribution. PCA with varimax rotation was used to extract factors. The Kaiser-Meyer-Olkin(KMO) Measure of sampling Adequacy (MSA) greater than 0.6 for individual as well as the full set of items was used to check the appropriateness of the PCA [13]. The MSA was 0.92 for the full set of items and >0.60 for all the individual items in the analysis, indicating adequacy for the purpose of factor analysis with a significant Bartlett’s test of sphericity(p = <0.0001). We used an eigenvalue ≥1 and a scree plot to determine the number of factors to be extracted.

In the PCA all 28 items were entered on an equal footing into the analysis process. This analysis extracted six factors with eigenvalues greater than 1, which explained 61.8% of the total variance in the original items. However, the step-by-step implementation criteria in the analysis of the PCA resulted in three factors with 9 items that explained 77.7% of the variance. Variables were included in the factor analysis if they had a communality value ≥ 0.5 and had no complex structure (loading ≥0.4 on more than one factor).

To attain the goal of homogeneity or uni-dimensionality (interpretability) of items loading in each extracted factors rather than internal consistency per se, one item was removed from the analysis, in spite of its high loading (0.74) on factor 1. The internal consistency of the scale was assessed by computing Cronbach’s alpha and item–total correlation coefficients. Deletion of item 16 did improve the internal consistency coefficient of the total scale by only 0.008, so it was included in the analysis. Generalizability of the data to the population from which the sample was taken was examined using split-half validation after splitting the data set by random number seed.

## Results

The instrument was distributed to 492 students. Two participants could not complete the questionnaire as they could not read the national official language, Amharic. Four orthodox Christian students refused to complete the questionnaire because it requested information that they regarded as transgressing God’s law. A list-wise deletion resulted in the exclusion of 22 cases due to some item non response. Thus, factor analysis was conducted on the data from 464 participants, 82.8% of whom were males. The mean and standard deviation for the age of the respondents were 21.2 ±2.1 years.

The ethnic groups reflected were diverse including 37.5% Amhara, 17.4% Oromo, 7.6% Tigre, 7.1% Kamata, 6.5% Sidama, 4.9% Guraghe, 4.2% Wolayita, 4.2% Hadiya and 10.5% other (Silte, Konso, Gamo, Sheka, Kaffa, Tembaro and many others). Sixteen students did not report their ethnic category. The predominant religion of the participants was Orthodox (60.8%), followed by Protestant (27.4%), Muslim (8.4%), and other (1.8%). Eight participants did not indicate their religion.

Regarding class year, 25%, 45.7%, 19.8% and 9.5% of respondents were in their 1st, 2nd, 3rd and 4th year of university study, respectively. About 39% (181) of participants had already experienced sexual intercourse and 77.9% of that group had ever used condoms.

### Constructing the Ethiopian version of CUSES (CUSES-E)

The PCA analysis extracted six factors with eigenvalues greater than 1, which explained 61.8% of the total variance in the original items. However, adherence to the inclusion criteria for PCA with varimax rotation produced a 9-item scale with a three factor structure. The three components together explained 77.7% of the total variance in the items. The scale had an MSA of 0.75 with significant Bartlett’s test of Sphericity (p < 0.001). The factors were named Assertiveness (items 4, 5, 6, 13), Fear for partner rejection (items 16, 17, 18), and Intoxicant Control (items 24, 25). The first component explained 38.8% of variance in the items and the remaining two factors explained 25.5% and 13.4%, respectively. Assertiveness involved free discussion about condom utilization with a partner. Fear for partner rejection involved the negative impressions a new partner might have regarding condoms. Intoxicant Control involved the perceived confidence in using condoms, despite being intoxicated (Table 1).

**Table 1:** Rotated factor loading and factor structure of CUSES-E

**Items in each factor**	**Loading**
**Factor 1: Assertiveness**	
I feel confident in my ability to discuss condom usage with any partner I might have	0.80
I feel confident in my ability to suggest using condoms with a new partner	0.87
I feel confident I could suggest using a condom without my partner feeling “diseased”	0.85
I feel confident in my ability to persuade a partner to accept using a condom when we have sex	0.77
**Factor 2: Fear for partner rejection**	
I wouldn’t feel confident suggesting using condoms with a new partner because I would be afraid he or she would think I’ve had a homosexual experience	0.83
I wouldn’t feel confident suggesting using condoms with a new partner because I would be afraid he or she would think I have a STD	0.92
I wouldn’t feel confident suggesting using condoms with a new partner because I would be afraid he/she would think I thought they had STD	0.88
**Factor 3: Intoxicant control**	
I feel confident that I would remember to use a condom even after I have been drinking.	0.93
I feel confident that I would remember to use condom even if I were high	0.93

### Internal consistency and homogeneity of the scale

The overall internal consistency coefficient (Cronbach’s alpha) for the scale was 0.778. In addition, the reliability of the extracted scale was evaluated by examining inter-item correlations. The mean inter-item correlation of the scale was 0.313. A mean inter-item correlation ≥0.3 shows reliability of the scale [21,22]. The entire item to total correlations was more than 0.3 satisfying criteria for reliability/homogeneity of items [21] (Table 2).

**Table 2:** Item-total correlation and percentage of students agreeing with each item in CUSES-E

**Items**	**Correlation coefficient**	**Strongly agree**	**Agree**	**Undecided**	**Disagree**	**Strongly disagree**
I feel confident in my ability to discuss condom usage with any partner I might have	0.69	142(30.6)	131(28.2)	76(16.3)	47(10.1)	68(14.7)
I feel confident in my ability to suggest using condoms with a new partner	0.79	177(38.1)	120(25.9)	63(13.6)	42(9.0)	62(13.4)
I feel confident I could suggest using a condom without my partner feeling “diseased”	0.73	146(31.5)	139(30)	83(17.9)	48(10.3)	48(10.3)
I feel confident in my ability to persuade a partner to accept using a condom when we have sex	0.73	150(32.3)	150(32.3)	80(17.2)	35(7.6)	49(10.6)
I wouldn’t feel confident suggesting using condoms with a new partner because I would be afraid he or she would think I’ve had a homosexual experience	0.30	46(9.9)	49(10.5)	68(14.7)	116(25)	185(39.9)
I wouldn’t feel confident suggesting using condoms with a new partner because I would be afraid he or she would think I have STD	0.43	40(8.6)	46(9.9)	50(10.8)	136(29.3)	192(41.4)
I wouldn’t feel confident suggesting using condoms with a new partner because I would be afraid he/she would think I thought they had STD	0.39	49(10.6)	34(7.3)	46(9.9)	144(31.0)	191(41.2)
I feel confident that I would remember to use a condom even after I have been drinking.	0.69	77(16.6)	94(20.3)	147(31.7)	64(13.8)	82(17.7)
I feel confident that I would remember to use condom even if I were high	0.69	79(17.0)	80(17.2)	157(33.8)	60(12.9)	88(19.0)

#### Construct validity

**Convergent and discriminant validity** The convergent and discriminant validity of the scale was examined using a multi-trait matrix. All the convergent correlations were higher than the discriminant ones (cross-construct correlation of items) implying the presence of both convergent and discriminant validity (Table 3).

**Table 3:** Multitrait matrix (inter item correlation) of CUSES_E

**Assertiveness**					**Fear for partner rejection**			**Intoxicant control**	
**Items**	**4**	**5**	**6**	**13**	**16**	**17**	**18**	**24**	**25**
**4**	**1**								
**5**	**0.65**	**1**							
**6**	**0.56**	**0.71**	**1**						
**13**	**0.52**	**0.62**	**0.59**	**1**					
**16**	0.03	0.03	0.06	0.08	**1**				
**17**	0.07	0.11	0.09	0.14	**0.64**	**1**			
**18**	0.06	0.08	0.06	0.1	**0.56**	**0.76**	**1**		
**24**	0.32	0.4	0.34	0.38	−0.03	0	−0.01	**1**	
**25**	0.32	0.4	0.34	0.35	**-**0.03	0.02	−0.01	**0.85**	**1**

Calculation of the correlations among all the items of the scale indicated that there were high inter-correlations among the items of the same factor (mean inter-correlations were 0.61, 0.65, 0.85, respectively for factors 1, 2 and 3), suggesting convergent validity. The subscales had high internal consistency coefficients. While the internal consistency coefficient was 0.92 for the “intoxicant control” subscale, it was 0.86 for both the “assertive” and the “fear for partner rejection” subscales. Similarly the three extracted components (subscales) had adequate correlation with the total scale. The corresponding correlation coefficients for factors 1, 2 and 3 were 0.83, 0.54 and 0.62 respectively. All the correlations were significant at p value of 0.01.

To further support the construct validity of the scale, an independent *t*-test with unequal variance was used to compare condom use self-efficacy between those who ever had sex and those who had not. Accordingly, students who differed on measures of previous condom use as well as on sexual intercourse experience showed significant differences(P < 0.001) on this scale and two subscales (assertiveness and intoxicant control) in the anticipated direction, indicating evidence of this scale’s discriminant validity. However, fear for partner rejection subscale did not discriminate between condom users and non-condom users (Table 4).

**Table 4:** Comparison of the scale (CUSES-E) and sub-scale scores by selected variables

		**Total score**		**Assertive**		**Fear for partner rejection**		**Intoxicant**	
								**control**	
		**Mean**	***t *test**	**Mean**	***t *test**	**Mean score**	***t *test**	**Mean score**	***t *test**
				**Score**					
	Yes	25.4	6.1*	12.2	7.3*	8.5		4.7	
Ever sex	No	21.4		9.4		8.4	0.083	3.6	4.7*
Ever used Condom	Yes	12.7	4.2*	12.7	7.4*	8.7		5	
	No	9.4		9.4		8.3	0.76	3.7	4.2*

*Significant at a p Value <0.0001.

To test the generalizability of findings from a principal component analysis, we conducted a split-half validation analysis on two randomly split samples. The communalities, factor loadings and factor structure were the same on the analysis for each half and for the full data set. Therefore, we have evidence that the findings are generalizable and valid, because, in effect, the two analyses represent a study and replication (Table 5).

**Table 5:** Split-half validation of the CUSES-E

**Items**	**First half (n = 227)**		**Second half (n = 233)**	
	**Communality**	**Loading**	**Communality**	**Loading**
**A. Assertiveness**				
**I feel confident in my ability to:**				
Discuss condom usage with any partner I might have	0.60	0.76	0.70	0.83
Suggest using condoms with a new partner	0.75	0.84	0.83	0.89
I could suggest using a condom without my partner feeling “diseased”	0.70	0.83	0.77	0.86
Persuade a partner to accept using a condom when we have intercourse	0.69	0.78	0.62	0.77
**B. Fear for partner rejection**				
I wouldn’t feel confident suggesting using condoms with a new partner because I would be afraid he or she would think I’ve had a homosexual experience	0.65	0.79	0.73	0.85
I wouldn’t feel confident suggesting using condoms with a new partner because I would be afraid he or she would think I have STD	0.82	0.89	0.88	0.93
I wouldn’t feel confident suggesting using condoms with a new partner because I would be afraid he or she would think I thought they had a STD.	0.72	0.88	0.79	0.89
**C. Intoxicant control**				
I would remember to use a condom even after I have been drinking.	0.92	0.93	0.94	0.93
I would remember to use a condom even if I were high	0.91	0.91	0.94	0.94
**Total variance explained**	**75.8%**		**79.8%**	
**KMO for MSA**	**0.72**		0.71	

## Discussion

The purpose of this study was to construct a condom use self-efficacy scale appropriate to the Ethiopian context using items from the original scale, which was developed to evaluate the self-efficacy of English speaking college students in the U.S. Unlike the original scale [14], the current analysis extracted three factor structures. Moreover, the number and types of items loading on each scale were not one and the same, implying cultural differences.

The first factor, labeled Assertiveness, is consistent with the Assertive factor reported in studies conducted in Ghana [10] and U.S [14,16]. In those studies, items loading on the Assertive factor involved one’s confidence to suggest condom use to a partner. However, items loading on the Assertiveness factor in the current study went beyond merely suggesting condom use and incorporated the ability to persuade a partner to use condoms. The Assertive factor was not found in a study conducted among college students in America [8], suggesting cultural diversity. A notable difference was observed in the factor labeled Appropriation by Barkley and Burns [8,10]. Owing to the conservative religious and cultural environment in Ethiopia, this factor did not emerge in the current study, which suggests a strong social norm against the acquisition, possession and application of condoms. This is in line with an HIV behavioral surveillance survey, where one reason for not using condoms in Ethiopia was the fear of being seen buying or possessing condoms [11].

The second factor we identified in this study was interpreted as Fear for partner rejection because the items loading on this component indicate the negative impressions a partner might have upon suggesting the use of condoms. This dimension was in agreement with a factor labeled STDs in the Ghana and American studies. The number and type of items loading in this subscale were one and the same across those studies and the current one [8,10]. Unlike the other subscales, this subscale does have global uniformity, with the exception of the study by Brien et al [16]. The third factor found in this study, which we labeled Intoxicant Control, is consistent with the dimension Intoxicants by Brien et al [16] and Pleasure and Intoxicants by Asante and Doku [10]. Two items related to drinking alcohol (confidence in using a condom while drinking or being high) loaded in those two studies and the current one, suggesting similarity of the study sites with regard to alcohol consumption.

According to Bandura, there are four major sources of self-efficacy [6,23]: mastery experience, social modeling, verbal persuasion and physiological response. Mastery experience suggests that individuals gauge the effects of their experiences and that their interpretations of these effects help create their efficacy beliefs [24]. Simply put, individuals learn from their own personal experience where they have achieved control. The significantly higher CUSES score among students in the current study who ever had sex and used condoms is consistent with the mastery experience concept of Bandura.

Bandura states that verbal persuasion originates not only from others but also from self-instruction [23]. The Assertiveness dimension of the extracted scale can be interpreted as a verbal persuasion component of self-efficacy, because the items loading on the subscale deal with one’s confidence in persuading a partner to use condoms.

The Fear for partner rejection factor is suggestive of Bandura’s physiological feedback component of self-efficacy, as was implied in the studies of Barkley and Burns [8]. According to Bandura [23] people can gauge their confidence by the emotional state they experience when contemplating an action. In the current study, a person’s fear of being wrongly perceived by a partner when suggesting condom use is consistent with the suggestion that emotional state plays an important role. Therefore, the extraction of factors indicated in Bandura’s sources of self-efficacy provides further support for the conceptual adequacy of the scale.

Though the current scale showed a somewhat different factor structure than previous studies [8,10,16], the scale appears to be highly reliable and valid. An inter-item correlation of 0.313 and a Cronbach’s alpha of 0.778 both provide evidence for the internal consistency of the scale [21].

The correlation matrix showed higher convergent correlations over the discriminant ones, which provides evidence for both types of validity. Like a similar study [10], the CUSES-E is associated with actual condom use in the Ethiopian context. However, unlike the two subscales (assertiveness and intoxicant control), fear for partner rejection subscale did not discriminate between condom users and non-condom users. Thus, further studies are needed to check suitability of this subscale in Ethiopia.

In the split sample validation, the communalities, factor loadings, and factor structure were the same for each half and for the full data set, suggesting that the new scale is generalizable and valid. However, the fact that our analysis like the Barkley and Burns study [8] left many items unassigned could be an evidence for limited importance of the items in the original scale which were developed in very different cultural context to the Ethiopian context. Therefore, future research to develop a suitable scale in concordance with the local vernacular using a prior qualitative study is needed.

A notable strength of this study is that study participants from diverse ethnic groups were selected using a probability sampling method. It must be noted, however, that students absent from class at the time of data collection were excluded as the study setting was a class room. Another notable limitation is that ever condom use was measured for every sex encounter irrespective of the type of sex partner. The results of this study should also be interpreted in accordance with the non response observed in this study.

## Conclusion

The finding that a unique dimension emerges when using an Ethiopian population suggests that CUSES should be validated to local contexts before application. The CUSES-E is valid, reliable and replicable. Therefore, health cadres and researchers in Ethiopia can apply this scale to promote condom utilization to Ethiopian school youths. However, future research to develop a suitable scale (highly valid and reliable) in concordance with the local vernacular using a prior qualitative study is needed.

## Abbreviations

CUSES: Condom use self-efficacy scale; CUSES-E: Condom use self-efficacy scale for Ethiopia; HIV: Human immunodeficiency syndrome; PCA: Principal component analysis; MSA: Measures of sampling adequacy.

## Competing interests

The authors declare that they have no competing interests.

## Authors’ contribution

DS: conceived the study, formulated research questions and design, ET: drafted the manuscript, DS and ET: reviewed the manuscript for intellectual content. DS and ET: coordinated data collection, analyzed and interpreted the data, read and approved the final manuscript.

## Pre-publication history

The pre-publication history for this paper can be accessed here:

http://www.biomedcentral.com/1472-698X/13/22/prepub
